# Antibacterial activities of selected edible plants extracts against multidrug-resistant Gram-negative bacteria

**DOI:** 10.1186/1472-6882-13-164

**Published:** 2013-07-10

**Authors:** Doriane E Djeussi, Jaurès AK Noumedem, Jackson A Seukep, Aimé G Fankam, Igor K Voukeng, Simplice B Tankeo, Antoine HL Nkuete, Victor Kuete

**Affiliations:** 1 Department of Biochemistry, Faculty of Science, University of Dschang, Dschang, Cameroon; 2 Department of Organic Chemistry, Faculty of Science, University of Dschang, Dschang, Cameroon

**Keywords:** Antibacterial, Multi-drug resistant bacteria, Dietary plants

## Abstract

**Background:**

In response to the propagation of bacteria resistant to many antibiotics also called multi-drug resistant (MDR) bacteria, the discovery of new and more efficient antibacterial agents is primordial. The present study was aimed at evaluating the antibacterial activities of seven Cameroonian dietary plants (*Adansonia digitata, Aframomum alboviolaceum, Aframomum polyanthum, Anonidium. mannii, Hibiscus sabdarifa, Ocimum gratissimum* and *Tamarindus indica*).

**Methods:**

The phytochemical screening of the studied extracts was performed using described methods whilst the liquid broth micro dilution was used for all antimicrobial assays against 27 Gram-negative bacteria.

**Results:**

The results of the phytochemical tests indicate that all tested extracts contained phenols and triterpenes, other classes of chemicals being selectively present. The studied extracts displayed various degrees of antibacterial activities. The extracts of *A. digitata, H. sabdarifa, A. polyanthum, A. alboviolaceum* and *O. gratissimum* showed the best spectra of activity, their inhibitory effects being recorded against 81.48%, 66.66%, 62.96%, 55.55%, and 55.55% of the 27 tested bacteria respectively. The extract of *A. polyanthum* was very active against *E. aerogenes* EA294 with the lowest recorded minimal inhibitory concentration (MIC) of 32 μg/ml.

**Conclusion:**

The results of the present work provide useful baseline information for the potential use of the studied edible plants in the fight against both sensitive and MDR phenotypes.

## Background

Pathogenic bacteria have always been considered as a major cause of morbidity and mortality in humans. Even though pharmaceutical companies have produced a number of new antibacterials in the last years, resistance to these drugs has increased and has now became a global concern [1]. The global emergence of multi-drug resistant (MDR) bacteria is increasingly limiting the effectiveness of current drugs and significantly causing treatment failure [2]. Bacterial resistance to chemically unrelated antimicrobial agents is public health concern [3] and may be caused by over-expression of MDR efflux pumps [4]. In Gram-negative bacteria, the effect of the efflux pumps in combination with the reduced drug uptake (due to the presence of a double membrane barrier) is responsible for the high inherent and acquired antibiotic resistance often associated with this group of organisms [5]. Among Gram-negative bacteria, many of these MDR efflux pumps belong to the RND (resistance-nodulation-cell division) type family of tripartite efflux pumps.

Due to the increase of resistance to antibiotics, there is a pressing need to develop new and innovative antimicrobial agents. Among the potential sources of new agents, plants have long been investigated. Because, they contain many bioactive compounds that can be of interest in therapeutic. Because of their low toxicity, there is a long tradition of using dietary plants in the treatment of infectious disease in Cameroonian folk medicine. Consequently, we focused one of the objective of our research group at investigating the antibacterial potentials of such plants against MDR phenotypes. In previous studies we demonstrated the antimicrobial activity of many Cameroonian dietary plants against MDR bacteria [6-9]. In our continuous search of the antibacterial activities of Cameroonian edible plants, we designed the present work to determine the activity of seven selected Cameroonian dietary plants (*Adansonia digitata, Aframomum alboviolaceum, Aframomum polyanthum, Anonidium mannii, Hibiscus sabdarifa, Ocimum gratissimum* and *Tamarindus indica*) against MDR Gram- negative bacteria.

## Methods

### Plant materials and extraction

The herbal sample consisted of seven different Cameroonian dietary plants namely the leaves of *Adansonia digitata* and *Anonidium mannii, the* rhizomes of *Aframomum alboviolaceum, Aframomum polyanthum,* the whole plants of *Hibiscus sabdarifa* and *Ocimum gratissimum,* and the fruits of *Tamarindus indica.* The plants were purchased from markets in the West region of Cameroon in January 2011. They were further identified at the National Herbarium (Yaoundé, Cameroon) where the voucher specimens were deposited under reference numbers (Table 1). Each plant was dried at room temperature and the powdered material was then weighed (300 g), soaked in 1 L of methanol (MeOH) for 48 h and filtered using Whattman N^o^1 filter paper. The filtrate obtained was concentrated under reduced pressure (at 68°C) in a rotary evaporator to obtain the crude extract. The crude extracts were kept at 4°C until further uses.

**Table 1:** Information on the plants used and report on evidence of their activities

Species (family); Voucher number*	**Traditional uses**	**Parts used traditionally**	**Bioactive or potentially bioactive components**	**Bioactivities**
***Adansonia digitata *(Malvaceae);** 42417/HNC	Analgesic, anti-diarrheal, smallpox, rubella [10], antipyretic, fever, dysenteria, anti-inflammatory, astringent [11]	Pulps, Fruits, leaves, Pip, Bark	/	Ethanol and aqueous extract: Ec [12] Sa, Se, STm, Pa [13]; [14,15] Hs [11].
***Aframomum alboviolaceum*** (Zingiberaceae); 34888/HNC	Diuretic, anthelmintic, fever, antiparasitic [16].	Roots	Methyl (E)-14Ksi,15-epoxylabd-8(17),12-dien-16-oate; (E)-labda-8(17),12-diene-15,16-dial and (E)-8beta,17-epoxylabd-12-ene-15,16-dial [17]	*Hc*[18].
***Aframomum polyanthum (Zingiberaceae)*** 3981/SRFK	/	Fruits	Aframodial [19].	Sa, Scp, Ha, Cu [19].
***Anonidium mannii (Annonaceae);*** 1918/SRFK	Spider and snake bites, bronchitis, dysenteria, gastroenteritis [20], syphilis, [21]; diarrhea, malaria [22].	Stem	Prenylatedbisindole [23].	/
		Bark		
		Leaves		
***Hibiscus sabdarifa *(Malvaceae);** 42795/HNC	Diuretic, stomachic, laxative, aphrodisiac, antiseptic, astringent, cholagogue, sedative, hypertension and other cardiac diseases [24].	Flowers	Protocatechic acid, [25]; [26], hydroxycitricacid.	Ethanol, methanol and aqueous extracts:Ec, Pa, Kp, Hi, Sa, Spy, Sp, [27]. Methanolic extract: Bs, Ml, Sm, Cs, Bc [24].
***Ocimum gratissimum *(Lamiaceae);** 42738/HNC	Respiratory tracts diseases, diarrhea, anti-hypertensive, malaria [28].	leaves, Roots, Buds	(β-caryophyllene,γ-terpinène, (Z)-α-bisabolene, thymol, p-cymene, eugenol, limonène, α-terpinolene, α-terpinéol [29].	Essential oil: Af, AB_1,_ Hc [30]Ethanol extract: Ec, Sa [31].
***Tamarindus indica *(Caesalpiniaceae);** 26326/SRFC	Fever, gastric ulcer, diarrhea, jaundice [32], conjunctivitis, hemorrhoid, astringent, asthma, eye inflammation [33].	Fruits Bark	/	Ethanol and aqueous extracts: Ec [12]

*(HNC): Cameroon National Herbarium; (SRFC): *Société des Réserves Forestières du Cameroun; AB*_*1*_: *Aflatoxin*_;_ Af; *Aspergillus flavus*; Bc: *Bacillus cereus*; Bs: *Bacillus stearothermophilus*; Cu: *Candida ufilis;* Cs: *Clostridium sporogenes*; Ec: *Escherichia coli*; Ha: *Hansenula anomala;* Hc: *Haemonchus contortus;* Hi: *Haemophilus influenza;* Kp: *Klebsiella pneumoniae*; Ml: *Micrococcus luteus*; Pa: *Pseudomonas aeruginosa*; Sa: *Staphylococcus aureus*; *Sc: Saccharomyces cerevisiae*; Se: *Staphylococcus epidermidis*; Scp: *Schizosaccharomyces pombe;* Sm: *Serratia mascences*; STm: *Streptococcus mutans;* Sp: *Streptococcus pneumoniae*; Spy: *Streptococcus pyogenes*; (/): not documented.

### Preliminary phytochemical screening

The plant materials were screened for the presence of different classes of secondary metabolites including alkaloids, flavonoids, phenols, saponins, tannins, anthocyanins, anthraquinones, sterols, and triterpenes using previously described methods [34].

### Bacterial susceptibility determinations

The minimal inhibitory concentrations (MICs) of the seven plant extracts were determined using a rapid *p*-Iodonitrotetrazolium chloride (INT; Sigma-Aldrich, St Quentin Fallavier, France) colorimetric assay [35,36]. Briefly, the test samples were first dissolved in dimethylsulfoxide (DMSO, Sigma-Aldrich)-Mueller Hinton Broth (MHB; Sigma-Aldrich). The solution obtained was then added to MHB and serially diluted two fold (in a 96-well microtilter plate). One hundred microliters of inoculums (1.5× 10^6^ CFU/ml) prepared in MHB were then added. The plates were covered with a sterile plate sealer and then agitated with a shaker to mix the contents of the wells and incubated at 37°C for 18 h. The final concentration of DMSO was less than 2.5%, and thus did not affect the microbial growth. Wells containing MHB, 100 μl of inoculum, and DMSO at a final concentration of 2.5% served as the negative control (this internal control with DMSO 2.5% was systematically added). Chloramphenicol (Sigma-Aldrich) was used as reference antibiotic. The MICs of each extract were detected after 18 h of incubation at 37°C following addition of 40 μl INT (0.2 mg/ml) and incubation at 37°C for 30 min. Viable bacteria reduced this yellow dye to pink. The MIC of each sample was defined as its lowest concentration that prevented this change and then resulted in the complete inhibition of microbial growth. The Minimum Bactericidal Concentration (MBC) was determined by sub-culturing samples from the wells with concentrations above the MIC on new plates of Mueller Hinton broth (MHB). The MBC was considered as the lowest concentration of the extract associated with no bacterial culture.

Each assay was performed three independent times in triplicate. In case where they were different, the MIC or MBC were taken as the most frequently occurring values. Chloramphenicol was tested alone and in the presence of Phenylalanine arginine-*ß*-naphtylamide (PAßN) at a final concentration of 30 μg/ml, as described previously [37].

## Results

### Phytochemical analysis

Freshly prepared extracts were subjected to a preliminary phytochemical screening for various constituents. The results (Table 2) revealed the presence of phenols, polyphenols and triterpenes. Anthraquinones were not detected in any of the extracts while anthocyanins were found only in the extracts of the genus *Aframomum (A. alboviolaceum* and *A. polyanthum).*

**Table 2:** Parts used, extraction yields, and phytochemical composition of the plant extracts

**Extracts**	***A. digitata***	***A. alboviolaceum***	***A. polyanthum***	***A. mannii***	***H. sabdarifa***	***O. gratissimum***	***T. indica***
**Parts used**	**Leaves**	**Fruits**	**Fruits**	**Leaves**	**Twigs**	**Twigs**	**Fruits**
Yield* (%)	**12.17**	**6.45**	**3.23**	**3.39**	**4.94**	**4.75**	**37.98**
Alkaloids	-	+	-	+	+	+	+
Anthocyanines	-	+	+	-	-	-	-
Anthraquinones	-	-	-	-	-	-	-
Flavonoids	-	+	-	-	+	-	+
Phenols	+	+	+	+	+	+	+
Polyphenols	+	+	+	+	+	+	+
Saponines	+	-	+	+	+	-	+
Tannins	+	-	-	+	-	+	-
Sterols	+	-	-	+	+	+	+
Triterpenes	+	+	+	+	+	+	+

(+): Present; (−): Absent; * yield calculated as the ratio of the mass of the obtained methanol extract/mass of the plant powder.

### Antibacterial activity of the plant extracts

The antibacterial activity of the plant extracts are depicted in Table 3. The results indicated that the plants extracts showed antibacterial activities at variable degrees against MDR bacteria, with MICs values varying from 32 to 1024 μg/ml. Extracts of *A. digitata* displayed the most important spectrum of activity, its inhibitory effects being observed against 81.48% of the bacterial strains, followed by the extracts of *H. sabdarifa* (66.66%)*, A. polyanthum (*62.96%)*, A. alboviolaceum (*55.55%) and *O. gratissimum* (55.55%). The extract of *A. polyanthum* showed the highest activity against *E. aerogenes* EA294 with a MIC value of 32 μg/ml. The extracts of *T. indica and A. mannii* did not show antibacterial activity against the majority of the bacteria tested, their inhibitory effect being noted against 6/27 (22.22%) and 7/27(25.92%) bacterial strains tested respectively. The microorganisms of the species *P. aeruginosa* (PA01 and PA124), known for their multi-resistance to drugs, were resistant to all the plant extracts tested in this work (with MIC > 1024 μg/ml).

**Table 3:** Minimal inhibitory concentration (MIC), minimal bactericidal (MBC) and MBC/MIC ratios of the plant extracts and CHL on the studied bacterial species

**Bacteria**		**Extracts and et antimicrobial parameters (MIC et MBC in μg/ml)**											
		***Adansonnia digitata***			***Aframomum alboviolaceum***			***Aframomum polyanthum***			***Anonidium mannii***		
		**MIC**	**MBC**	**MBC/MIC**	**MIC**	**MBC**	**MBC/MIC**	**MIC**	**MBC**	**MBC/MIC**	**MIC**	**MBC**	**MBC/MIC**
*E. coli*	ATCC8739	1024	-	-	-	-	-	1024	**-**	**-**	**-**	**-**	**-**
	ATCC10536	-	-	-	-	-	-	512	**-**		**-**	**-**	**-**
	AG100	512	-	-	1024	-	-	-	**-**	**-**	**-**	**-**	**-**
	AG100A	128	-	-	1024	-	-	-	**-**	**-**	**-**	**-**	**-**
	AG100A_TET_	1024	-	-	256	-	-	1024	**-**	**-**	1024	**-**	**-**
	AG102	256	512	2	1024	-	-	512	**-**	**-**	-	**-**	**-**
	MC4100	1024	1024	1	1024	-	-	-	**-**	**-**	512	**-**	**-**
	W311O	512	-	-	-	-	-	512	**-**	**-**	-	**-**	**-**
*E.aerogenes*	ATCC13048	128	512	4	512	-	-	-	**-**	**-**	-	**-**	**-**
	CM64	1024	-	-	-	-	-	1024	**-**	**-**	-	**-**	**-**
	EA27	-	-	-	1024	-	-	-	**-**	**-**	1024	**-**	**-**
	EA289	512	-	-	1024	-	-	1024	**-**	**-**	1024	**-**	**-**
	EA298	1024	1024	1	-	-	-	1024	**-**	**-**	-	**-**	**-**
	EA294	1024	-	-	256	-	-	32	512	16	-	**-**	**-**
*E. cloacae*	ECCI69	512	-	-	-	-	-	1024	-	-	1024	**-**	**-**
	BM47	1024	-	-	-	-	-	1024		-	-	**-**	**-**
	BM67	1024	-	-	1024		-	512	-	-	1024	**-**	**-**
***K. Pneumonia***	ATCC11296	512	-	-	-	-	-	256	1024	4	-	**-**	**-**
	KP55	128	256	2	512	1024	2	-	**-**	**-**	-	**-**	**-**
	KP63	1024	-	-	1024	-	-	-	**-**	**-**	-	**-**	**-**
	K24	512	-	-	512	-	-	1024	**-**	**-**	-	**-**	**-**
	K2	1024	-	-	512	-	-	1024	**-**	**-**	1024	**-**	**-**
*P. Stuartii*	ATCC29914	-	-	-	-	-	-	512	**-**	**-**	**-**	**-**	
	PS2636	1024	-	-	1024	-	-	1024	**-**	**-**	**-**	**-**	**-**
	PS299645	1024	-	-	-	-	-	-	**-**	**-**	**-**	**-**	**-**
*P. aeru-ginosa*	PA01	**-**	**-**	**-**	**-**	**-**	**-**	**-**	**-**	**-**	**-**	**-**	**-**
	PA124	**-**	**-**	**-**	**-**	**-**	**-**	**-**	**-**	**-**	**-**	**-**	**-**
**Bacteria**		***Hibiscus sabdarifa***			***Ocimum gratissimum***			***Tamarintus indica***			Chloramphenicol*		
		**MIC**	**MBC**	**MBC/MIC**	**MIC**	**MBC**	**MBC/MIC**	**MIC**	**MBC**	**MBC/MIC**	**MIC**	**MBC**	**MBC/MIC**
*E. coli*	ATCC8739	-	-	-	512	-	-	-	-	-	4	-	-
	ATCC10536	-	-	-	1024	-	-	-	-	-	2	128	64
	AG100	1024	-	-	-	-	-	-	-	-	4 (2)	256 (32)	64 (16)
	AG100A	512	-	-	-	-	-	-	-	-	2	64	32
	AG100A_TET_	1024	-	-	-	-	-	-	-	-	64 (8)	256 (64)	4 (8)
	AG102	1024	-	-	1024	1024	1	-	-	-	8	-	-
	MC4100	1024	1024	1	512	-	-	1024	-	-	64	-	-
	W311O	1024	1024	1	-	-	-	512	-	-	4	32	8
*E.aerogenes*	ATCC13048	1024	-	-	1024	-	-	-	-	-	8	128	16
	CM64	1024	-	-	1024	-	-	-	-	-	256 (64)	- (64)	- (1)
	EA27	1024	-	-	-	-	-	-	-	-	256 (32)	512 (256)	2 (8)
	EA289	1024	-	-	512	-	-	-	-	-	512 (32)	512 (128)	1(4)
	EA298	512	-	-	512	-	-	1024	1024	1	128 (64)	- (64)	- (1)
	EA294	1024	-	-	1024	-	-	-	-	-	4	16	4
*E. cloacae*	ECCI69	-	-	-	512	-	-	1024	-	-	512	-	-
	BM47	-	-	-	-	-	-	-	-	-	512	-	-
	BM67	256	1024	4	-	-	-	1024	-	-	256	-	-
*K. Pneumonia*	ATCC11296	1024	-	-	1024	-	-	-	-	-	4	512	128
	KP55	512	-	-	512	-	-	-	-	-	128	128	1
	KP63	-	-	-	1024	-	-	-	-	-	64 (16)	- (256)	- (16)
	K24	1024	-	-	1024	-	-	-	-	-	16 (1)	- (64)	- (64)
	K2	512	-	-	-	-	-	1024	-		32	-	-
*P. Stuartii*	ATCC29914	-	-	-	-	-	-	-	-	-	8	128	16
	PS2636	-	-	-	128		-	-	-	-	32	256	8
	PS299645	512	-	-	-	-	-	-	-	-	16	512	32
*P. aeru-ginosa*	PA01	-	-	-	-	-	-	-	-	-	16 (8)	- (256)	- (32)
	PA124	-	-	-	-	-	-	-	-	-	32 (16)	- (−)	- (−)

(−): >1024 μg/ml for extracts and >512 μg/ml for chloramphenicol and not calculated for MBC/MIC.

*(): for chloramphenicol in the presence of PABN.

Some of the studied extracts showed bactericidal effects on few numbers of bacteria. These effects were observed with the crude extracts of *A. digitata*, against *E. coli* MC4100 and *K. pneumoniae* KP55 with the ratios minimal bactericidal versus minimal inhibitory concentrations (MBC/MIC) equal to 1 and 2 respectively. For *A. polyanthum’s* extract*,* the ratio MBC/MIC was equal to 2 on *K. pneumoniae* KP55. *O. gratissimum* also showed ratios MBC/MIC equal to 1 on *E. coli* AG 102. The crude extract of *H. sabdarifa* was also bactericidal against *E. coli* MC4100 and W3110 and against *E. cloacae* BM67 with the ratio equal to 1; 1 and 4 respectively. Chloramphenicol used as reference antibiotic showed variable inhibitory activity on different strains of bacteria with MIC values ranging from 2 to 512 μg/ml. These activities of chloramphenicol was bacteristatic on the majority of bacteria (MBC/MIC > 4) and in some cases, its MICs were equal to those obtained with some plant extracts (*A. digitata* on *K. pneumoniae* KP55, *H. sabdarifa* on *E. cloacae* BM 67 and *O. gratissinum on E. cloacae ECCI69*).

## Discussion

Each of the extract tested in the present study displayed antibacterial activity on at least 6 of 27 bacterial strains tested. However differences were observed between antibacterial activities of the extracts. These differences could be due to the differences in the chemical composition of these extracts as the secondary metabolites of plants have many effects including antibacterial and antiviral properties [9,38]. The overall data of this study were in accordance with previous results. Apart from the phytochemicals found in *A. digitata* extract, previous studies showed the presence of an alkaloid namely adansonin [15]. The antibacterial activity of the aqueous and ethanol extracts of this plant has already been reported against *E. coli*[12]. Therefore, the inhibitory activity found herein against reference and multi-resistant strains of *E. coli* as well as other Gram-negative species is complementary to Yagoub’s [12] report.

Phytochemical screening results of *H. sabdarifa* was in accordance with the results previously obtained [24]. This latter suggested that the presence of alkaloids (which interfere with cell division) in *H. sabdariffa* could account for its antimicrobial activity. They demonstrated that methanol extract of *H. sabdarifa* possess inhibitory activities against *E. coli, P. aeruginosa* and *S. aureus.* In this report, the antibacterial activity was not observed against *P. aeruginosa*, but the results obtained herein are not in contradiction with those previously reported since the previous MIC of 1300 μg/ml was higher than the highest concentration used in this work. The results of the present work also bring additional data on the antibacterial activity of *H. sabdarifa*, since we report for the first time its activity against *E. aerogenes, P. stuartii* and *K. pneumoniae.*

To the best of our knowledge, phytochemical composition of *A. alboviolaceum* and *A. polyanthum* is described here for the first time. The different phytochemicals found here should then explain its antibacterial activity against different bacterial strains tested. The plants of the genus *Aframomum* was already found to possess flavonoids, diterpenoids and arylalkaloids which could explain their antibacterial activity [39].

All the phytochemical constituent found in the extract of *O. gratissinum* was previously reported by Akinmoladun *et al.*[40] who also found flavonoids in the same extract. Nevertheless, the antibacterial activity of this extract is in agreement with the findings of Obinna *et al.*[31] who showed the inhibitory activity of *O. gratissimum* against *E. coli* and *S. aureus.* Moreover the present work brings additional information of the antibacterial activities of this plant against multi-resistant bacteria.

Previous reports showed good antibacterial effect of *T. indica* against *E. coli* strains isolated from urine and water samples. Another plant of the present work namely *A. manni* is used traditionally for treatment of different ailments including different infectious diseases like gastroenteritis and syphilis. PAßN, is a potent inhibitor of the RND efflux systems is especially active on AcrAB-TolC and MexAB-OprM. The wide range enhancement (on all the strains) of the antibacterial activity by PAβN observed herein with chloramphenicol confirmed that an active efflux system expressed by tested bacteria is responsible for their resistance to chloramphenicol. The wide substrate specificity of these pumps could allow them to provoke extrusion of various active antibacterial compounds, preventing their inhibitory effects [9]. Therefore, the low antibacterial activities of these plants shown in the present work should thus be due to the resistance of bacteria strains tested (see Additional file 1: Table S1). The contrast between high number of secondary metabolite classes found in these extracts reinforces the idea that the detection of the classes of phytochemicals in plants is not a guarantee for a good antibacterial properties [9]. A sample is bactericidal when the ratio MBC/MIC ≤ 4 and bacteriostatic when this ratio is >4 [9]. It therefore appeared that bactericidal effects were obtained with the extract from *A. alboviolaceum, T. indica* and *O. gratissimum* against 1 of the 27 tested bacterial strains and *A. digitata* against 5/27 (Table). No bactericidal activity was obtained with *A. mannii* extract on all the studied bacteria. This shows that the studied extract mostly exhibited bacteriostatic effects.

## Conclusion

The results of the present study support the traditional use of the studied plants in the treatment of bacterial infections. They also provide an important basis for the use of methanol extract of the edible plants used to control infectious diseases caused by Gram-negative bacteria including MDR strains.

## Abbreviations

ATCC: American Type Culture Collection; CFU: Colonies forming unit; CHL: Chloramphenicol; DMSO: Dimethylsulfoxyde; INT: p-iodonitrotetrazolium chloride; MDR: Multidrug Resistant; MHB: Mueller Hinton Broth; MIC: Minimal Inhibitory Concentration; PAßN: Phenylalanine Arginine *ß*-Naphthylamide; RND: Resistance Nodulation-cell Division.

## Competing interests

The authors declare that they have no competing interest.

## Authors’ contributions

DED, JAKN, AGF, IKV, SBT, AHLN and AJS carried out the study; VK designed the experiments, supervised the work; JAKN and VK wrote the manuscript; VK provided the bacterial strains; All authors read and approved the final manuscript.

## Pre-publication history

The pre-publication history for this paper can be accessed here:

http://www.biomedcentral.com/1472-6882/13/164/prepub

## Supplementary Material

Additional file 1: Table S1 Bacterial strains and features. Click here for file
